# Characteristic Metabolic Alterations Identified in Primary Neurons Under High Glucose Exposure

**DOI:** 10.3389/fncel.2018.00207

**Published:** 2018-07-17

**Authors:** Liangcai Zhao, Minjian Dong, Dan Wang, Mengqian Ren, Yongquan Zheng, Hong Zheng, Chen Li, Hongchang Gao

**Affiliations:** ^1^School of Pharmaceutical Sciences, Wenzhou Medical University, Wenzhou, China; ^2^Department of Pharmacy, Women’s Hospital, Medicine of School, Zhejiang University, Hangzhou, China

**Keywords:** neuron, ATP, high glucose, nuclear magnetic resonance, metabonomics

## Abstract

Cognitive dysfunction is a central nervous system (CNS) complication of diabetes mellitus (DM) that is characterized by impaired memory and cognitive ability. An in-depth understanding of metabolic alterations in the brain associated with DM will facilitate our understanding of the pathogenesis of cognitive dysfunction. The present study used an *in vitro* culture of primary neurons in a high-glucose (HG) environment to investigate characteristic alterations in neuron metabolism using nuclear magnetic resonance (NMR)-based metabonomics. High performance liquid chromatography (HPLC) was also used to measure changes in the adenosine phosphate levels in the hippocampal regions of streptozotocin (STZ)-induced diabetic rats. Our results revealed significant elevations in phosphocholine and ATP production in neurons and decreased formate, nicotinamide adenine dinucleotide (NAD^+^), tyrosine, methionine, acetate and phenylalanine levels after HG treatment. However, the significant changes in lactate, glutamate, taurine and myo-inositol levels in astrocytes we defined previously in astrocytes, were not found in neurons, suggested cell-specific metabolic alterations. We also confirmed an astrocyte-neuron lactate shuttle between different compartments in the brain under HG conditions, which was accompanied by abnormal acetate transport. These alterations reveal specific information on the metabolite levels and transport processes related to neurons under diabetic conditions. Our findings contribute to the understanding of the metabolic alterations and underlying pathogenesis of cognitive decline in diabetic patients.

## Introduction

Diabetes mellitus (DM) is a systemic metabolic disease that is characterized by chronic hyperglycemia, hyperlipidemia and other alterations, including insulin resistance or insufficient production (Hardigan et al., 2016). Cognitive impairment is one diabetic complication that reduces patients’ quality of life (Duarte et al., 2007; Ehehalt et al., 2009). The known mechanisms of this complication include oxidative stress (Yi et al., 2011), advanced glycation end products (Wang et al., 2009) and mitochondrial dysfunction (Ye et al., 2011). Diabetes-related cognitive impairment also results from disrupted insulin signaling and glucose homeostasis in the brain (Reagan et al., 2008) or diabetes-associated microvascular changes (Wessels et al., 2008). All of these factors are critical factors in the development of cognitive decline in diabetic patients. However, the pathogenic mechanism and metabolic alterations involved in this decline have not been clarified.

Metabonomics is a system biology technology that focuses on investigations of low molecular weight substances under pathophysiological conditions (Nagana Gowda and Raftery, 2017). Metabonomics has been extensively used in the identification of global metabolic information from various biological samples (Gil et al., 2015; Chen et al., 2016; Zhao et al., 2016), especially the metabolic characteristics related to diseases (Duarte et al., 2009). Nuclear magnetic resonance (NMR) spectroscopy is a commonly used analytical method for the detection of a series of cerebral substances, including energy metabolites, neurotransmitters, membrane metabolites and antioxidants (Soares and Lei, 2017). NMR-based metabonomic studies have undoubtedly improved our understanding of the mechanisms involved in the pathogenesis of neurologic diseases (Alonso et al., 2014). By which we found an increase in glycolytic activity, inhibition of the tricarboxylic acid cycle (TCA cycle) and dysfunction in glutamate-glutamine shuttling between neurons and astrocytes in different brain regions (Zheng et al., 2016, 2017c). ^13^C NMR technology with [1-^13^C]-glucose and [2-^13^C]-acetate substrates also revealed impaired neuronal activity, enhanced astrocytic activity and lower levels of ^13^C labeling in TCA intermediates in neurons in the diabetic rats (Wang et al., 2015; Zheng et al., 2017a). These results demonstrated differences between neurons and astrocytes, especially under hyperglycemic conditions.

Primary cells were cultured under high glucose (HG) exposure as an *in vitro* diabetic environment and to further identify characteristic changes in neurons and astrocytes. Astrocytes exhibited increased levels of lactate production and release, increased production of osmolyte and excessive consumption of certain amino acids (Wang et al., 2018). The present study used a primary neuron culture to identify the metabolic features of neurons during hyperglycemia. The present study: (i) investigated the metabolic features of neurons, i.e., absorption and release of metabolites under physiological conditions; (ii) measured metabolic changes in neurons under HG conditions; and (iii) clarified the respective metabolic features of neurons and astrocytes under HG exposure.

## Materials and Methods

### Reagents

Neurobasal Medium, Dulbecco’s Modified Eagle’s Medium (DMEM), fetal bovine serum (FBS), Hank’s balanced salt solution (HBSS), B-27 Serum-Free Supplement, L-glutamine and 0.25% trypsin-EDTA were purchased from Gibco, Langley, OK, USA. Cell culture flasks, 6-well plates, 96-well plates and Pasteur pipettes were purchased from Corning Inc., Corning, NY, USA. Deuterium oxide (D_2_O), TMSP-2,2,3,3-D4 (D, 98%) and sodium-3-trimethylsilylpropionate (TSP) were purchased from Cambridge Isotope Laboratories, Tewksbury, MA, USA. D-(+)-glucose, streptozotocin (STZ) and ATP, ADP, AMP standards were purchased from Sigma Aldrich, St. Louis, MO, USA. Poly-D-lysine and the penicillin-streptomycin solution were purchased from Beyotime Biotechnology, China. The MAP2 antibody and goat anti-rabbit IgG-FITC antibody were obtained from Abcam, Britain and Santa Cruz Biotechnology, Santa Cruz, CA, USA, respectively.

### Animal Use and Procedures

Male Sprague-Dawley rats weighing 160–180 g and Sprague-Dawley rat pups were purchased from specific pathogen-free colonies from the Animal Laboratory Center of Wenzhou Medical University. Rats were housed in an environment with regulated temperature and humidity and a 12-h light-dark cycle with lights on at 08:00 a.m. Care and research procedures were performed in accordance with the ARRIVE Guidelines and the “Guide for the Care and Use of Laboratory Animals.” Procedures using rats were approved by the Institutional Animal Care and Use Committee of Wenzhou Medical University (document number: wydw2012-0083).

Adult rats were fasted for 12 h before being randomly injected intraperitoneally with STZ freshly prepared in citrate buffer (0.1 M, pH 4.5) using a single dose of 60 mg/kg body weight. Control rats were injected with the same volume of citrate buffer. Blood glucose concentrations were measured using a glucometer 2 days after STZ treatment, and rats with blood glucose greater than 16.70 mmol/l were defined as diabetic. Five (short term diabetes) or 15 (long term diabetes) weeks after STZ injection, rats were fasted overnight prior to euthanization via cervical decapitation. The hippocampus was dissected immediately, snap-frozen in liquid nitrogen and stored at −80°C until assayed.

### Cell Culture and Treatment

Primary neurons were cultured as previously described with some modifications (Kaech and Banker, 2006; Brewer and Torricelli, 2007). Briefly, cultures were prepared from Sprague-Dawley rat pups that were less than 1 day old. Cerebral cortical tissues were dissected under sterile conditions, and the meninges were removed under a dissecting microscope. Tissues were minced and digested using 0.25% trypsin for 10 min at 37°C, and the trypsin was neutralized using DMEM medium containing 10% FBS. The cell suspension was passed through a 200-micron mesh cell strainer to obtain single cell suspensions. Cells were plated on poly-D-lysine-coated Falcon flasks (75 cm^2^) at a density of 2 × 10^6^ cell/ml. Cells were cultured in a humidified incubator with 5% CO_2_ at 37°C (Thermo Fisher Scientific, Waltham, MA, USA) for 12 h of culture. Cells were washed once with HBSS, and the culture medium was replaced with fresh Neurobasal serum-free medium supplemented with B27 (1×), 2 mM glutamine, 100 U/ml penicillin and 0.1 mg/ml streptomycin. Cytosine arabinoside was added to a final concentration of 10 μM after 48 h in culture and maintained for 12 h to prevent astrocyte proliferation. Neurons were cultured for 4 days. The medium was changed twice weekly.

Neurons were seeded in 12 culture flasks (2 × 10^7^ cells/flask) and five 24-well plates (10^5^ cells/well) for intracellular and extracellular metabolic studies, respectively. Cells were cultured with 10 ml of Neurobasal medium in each flask and 1.1 ml Neurobasal medium in each well of the 24-well plate. Cells for HG treatment were washed once with HBSS, and the culture media was replaced with fresh Neurobasal medium containing 25 mM glucose for the control treatment or 50 mM for the HG treatment.

### Immunofluorescence

Neurons were cultured on coverslips, fixed in 4% paraformaldehyde for 45 min and permeabilized with 0.5% (v/v) Triton X-100 in PBS for 15 min. Nonspecific binding was blocked using 1% (w/v) bovine serum albumin for 1 h. Cells were incubated overnight in a humidified chamber at 4°C with primary antibody (1:1000, rabbit polyclonal to MAP2, Abcam, Britain) followed by fluorescent-conjugated secondary antibodies for 1 h under ambient temperature (1:250, goat anti-rabbit, Santa Cruz Biotechnology, Santa Cruz, CA, USA). Cell nuclei were stained with Hoechst (5 μg/ml) for 5 min. Fluorescent images were captured using a fluorescent microscope (Nikon, Japan).

### Cell Viability Assay

The viability of neurons was quantified using the MTT assay system. Briefly, neurons were plated into 96-well plates at a density of 5 × 10^4^ cells/well in 100 μl culture media. Neurons were washed once with HBSS when confluent, and the culture medium was replaced with fresh Neurobasal medium (control group) or medium containing 50 mM glucose (HG group). Neurons were exposed to HG for 72 h and treated with 20 μl MTT (5 mg/ml) for 4 h. The medium was removed carefully, and 100 μl DMSO was added to solubilize the formazan crystalline product. The OD values of the solubilized formazan were related to viable cells and detected at 570 nm with a reference wavelength at 630 nm using a microplate reader (Multiskan Mk3, Thermo Labsystems, Finland). Cell viability was expressed as the ratio of the treated groups to the control group. Each group contained six wells, and experiments were repeated in three biological replicates.

### Apoptosis and Nuclear Morphology Assay

Nuclear morphology was used to assess cells undergoing apoptosis (cells with condensed or fragmented nuclei; Gaspar et al., 2010; Xu et al., 2012; Liu et al., 2014). Cells were gently washed with PBS after treatment and incubated with 2 μg/ml fluorescent Hoechst dye (5 μg/ml) for 5 min at room temperature in the dark. Fluorescent images of nuclei were captured using a fluorescent microscope (Nikon, Japan). A total of six fields within each well were counted. Apoptosis was the proportion of apoptotic nuclei in each group.

### Reactive Oxygen Species (ROS) Measurement

Reactive oxygen species (ROS) production was measured with ROS assay kit (Beyotime, S0033) according to manufactures’ instruction. Briefly, following HG treatment, the medium was aspirated and cells were incubated with 10 μM fluorogenic probe dichloro-dihydro-fluorescein diacetate (DCFH-DA) in new media at 37°C for 20 min, then cells were washed twice with serum-free media. Cellular DCF fluorescence intensities were determined by fluorescence microscopy and the images were analyzed by ImageJ software (1.48v, US National Institutes of Health, Bethesda, MD, USA).

### Cellular Malondialdehyde (MDA) and Reduced Glutathione (GSH) Measurement

Cells were incubated with or without glucose (50 mM) for 72 h. Cellular Malondialdehyde (MDA) were measured according to the previously described method using a kit (Nanjing Jiancheng Bioengineering Institute, Nanjing, China), which is based on the principles in the previous study (Zhu et al., 2014). Briefly, MDA reacts with thiobarbituric acid (TBA) at 90–100°C and acidic condition. The reaction yields a pink MDA-TBA conjugate, which was measured at 532 nm using a Multi-Mode Microplate Readers (Spectra Max M5). The cellular MDA was expressed as nmol/mg protein. Cellular Glutathione (GSH) were measured according to the previously described method using a kit (Nanjing Jiancheng Bioengineering Institute, Nanjing, China). Briefly, reduced GSH reacts with dithiobis nitrobenzoic acid (DTNB) at room temperature for 5 min. The reaction yields a yellow substance, which was measured at 405 nm using a Multi-mode Microplate Readers (Spectra Max M5). The cellular GSH was expressed as μmol/g protein.

### Metabolite Extraction and ^1^H-NMR Data Acquisition

Neurons were harvested after rapid quenching at the end of HG stimulation, as described by Teng et al. (2009). Quenched cells were pelleted via centrifugation and rinsed with HBSS prior to transfer to a centrifuge tube. Cell pellets were resuspended in 450 μl of ice-cold CH_3_OH:CHCl_3_ (v/v 2:1) and sonicated on ice for 30 min. Ice-cold CHCl_3_:H_2_O (v/v 1:1; 450 μl) was added to form an emulsion, which was left on ice for 15 min prior to centrifugation at 12,000 rpm for 20 min (4°C). The upper phase was collected, lyophilized and stored at −80°C until NMR analysis (1 flasks/sample, *n* = 6/group).

After HG treatment, 1 milliliter of culture media from each well of plates was transferred to individual 15-ml centrifuge tubes, and 3 ml ice-cold CH_3_OH:CHCl_3_ (v/v 2:1) was added followed by 1 ml of ice-cold CHCl_3_. The tubes were mixed vigorously for 60 s on ice prior to centrifugation at 8000 rpm for 20 min at 4°C. The supernatants were collected, lyophilized and stored at −80°C for NMR analysis.

High-resolution ^1^H-NMR spectra of cell and media extracts were obtained using a Bruker AVANCE III 600 spectrometer operating at 600.13 MHz equipped with a triple resonance probe. The lyophilized samples were dissolved in 450 μl D_2_O and transferred into 5-mm NMR tubes. Spectra were acquired with an 8.0 s relaxation delay, 32 K data point, the acquisition time was 5.44 s, total of 2048 scans for intracellular samples and 256 scans for extracellular samples. A one-dimensional ZGPR pulse sequence was used to achieve satisfactory water suppression in aqueous extracts.

### Data Reduction and Multivariate Data Analysis

All NMR spectra were phase- and baseline-corrected using the Bruker Topspin 2.1 software (Bruker Biospin, Germany) and referenced to the methyl peak of lactate (CH_3_) at 1.33 ppm. The spectral region from 0.4 ppm to 10.0 ppm was subdivided with a size of 0.01 ppm for multivariate pattern recognition analysis and 0.0015 ppm for quantitative analysis. The region of approximately δ4.60–6.12 and δ2.17–3.16 was removed to eliminate artifacts related to residual water resonance and the HEPES solvent peak. The remaining spectral segments were normalized to the total sum of the spectral intensity to compensate for variations in total sample volume.

A multivariate statistical analysis was performed using the SIMCA 13.0 software package (Umetrics, Umeå, Sweden). Partial least-squares discriminate analysis (PLS-DA) were carried out for class discrimination and biomarker identification. Data were visualized by plotting the scores of the first two principal components (PC1 and PC2) to provide the most efficient 2D representation of the information, where the position of each point along a given axis in the scores plot was influenced by variables of metabolite. PLS-DA revealed differences in the composition of different groups, which were necessary to eliminate outliers and enhance the quality of the PCA model. Leave-one-out cross validation and permutation tests (200 cycles) were performed to assess the performance of PLS-DA. The parameters R2 and Q2 were computed to test the goodness of fit and model validity, where R2 is the fraction of the sum of square of the entire X’s explained by the model and Q2 represents the cross-validated explained variation with increasing reliability. Leave-one-out cross validation and permutation tests (200 cycles) were conducted to measure the robustness of the model obtained because of the small number of samples. If the plot shows that the Q2 regression line has a negative intercept, and all permuted R2 values on the left are lower than the original point on the right, then, the PLS-DA model built for different groups is robust and credible, an excellent model was indicated when these two parameters were close to 1.0 (Cloarec et al., 2005; Tian et al., 2016).

### HPLC Assay

Hippocampus samples were homogenized using 12% perchlorate. The mixture was centrifuged at 1000 *g* at 4°C for 15 min, and the supernatant was filtered and injected into a high-performance liquid chromatogram (HPLC) system. HPLC analysis was performed using the AccQ·Tag HPLC system (Waters, Milford, MA, USA) equipped with an Agilent C_18_ column (5 μm, 150 × 4.6 mm). The mobile phase consisted of 0.1 mM buffer and 1% methanol, and the injection volume was 20 μl. The flow rate was 1 ml/min, and the column temperature was maintained at 25°C. The identification of different adenine nucleotides was based on the retention times of known amounts of standards. The adenine nucleotides were quantified using the standard curve of standards in the chromatogram.

### Statistical Analysis

SPSS software (version 16.0) was used to evaluate significant differences between the control and HG groups. Data were analyzed using an independent sample *t*-test. *P* < 0.05 was considered statistically significant. The results are reported as the means ± SD. The correlation matrices of extracellular metabolite balances were calculated using Pearson correlation coefficients on normalized data. An absolute value of correlation coefficient |*r*| > 0.7 (*P* < 0.05) indicated a statistically significant relationship.

## Results

### High Glucose Exposure of Primary Cultured Neurons

**Figure 1:**
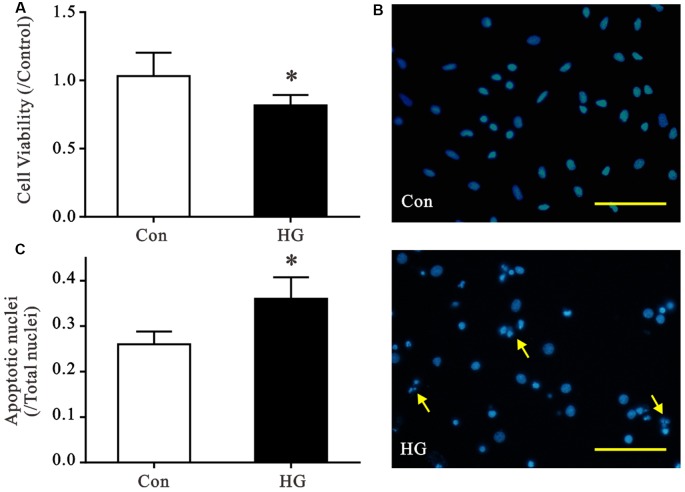
Effects of high glucose (HG) exposure on neurons. Primary neurons were exposed to control (CON) or 50 mM glucose for 72 h. **(A)** HG treatment significantly decreased cell viability in primary neurons compared with the control group. **(B,C)** HG treatment increased the proportion of condensed/fragmented nuclei (yellow arrow, Scale bar = 500 μm). Data presented as mean ± SD (*n* = 6). *p < 0.05 vs. control group by independent sample *t*-test.

We established primary cultures of rat neurons and verified purity using immunofluorescent staining. Most cells were positive for MAP2 staining, which suggests cell purity >90% (Supplementary Figure S1). Cell viability and nuclear morphology were evaluated using MTT and Hoechst staining, respectively, to determine the detrimental effects of HG (50 mM) on primary neurons (Figure 1). The viability of HG-treated neurons decreased significantly after 72 h of HG exposure (Figure 1A), and a remarkable increase in apoptotic nuclei was observed compared to control glucose levels (Figures 1B,C).

**Figure 2:**
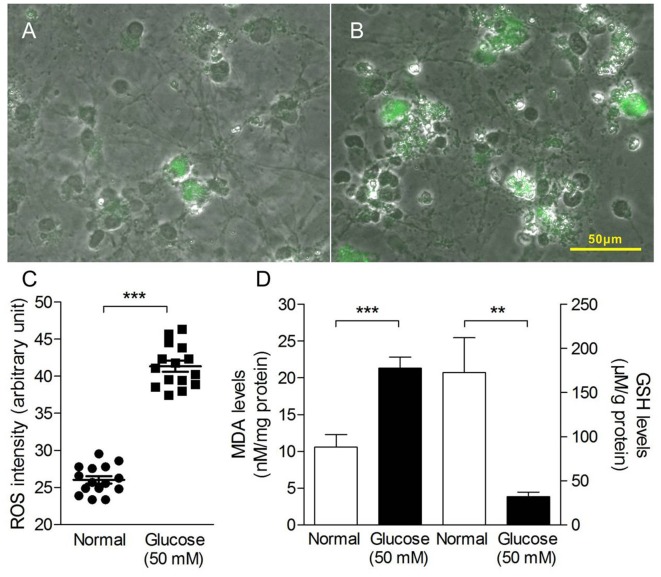
Oxidative stress production in primary neurons induced by HG treatment. Merged pictures of cell morphology and fluorescence images taken from primary neuron in the absence **(A)** or presence of glucose (50 mM, **B**) for 4 h and stained with dichloro-dihydro-fluorescein diacetate (DCFH-DA; 10 μM for 20 min). **(C)** Quantitative analysis of DCF fluorescence intensity as an indication of reactive oxygen species (ROS) levels in the HG-induced primary neuron cells. **(D)** The malondialdehyde (MDA) levels (left *y*-axis) and glutathione (GSH) levels (right *y*-axis) of HG-induced primary neuron cells for 72 h. Data presented as mean ± SD. **p < 0.01, ***p < 0.001 vs. control group by independent sample *t*-test.

Oxidative stress in the study was measured as production of ROS and MDA, GSH levels. As shown in Figure 2, 50 mM glucose significantly enhanced intracellular ROS levels (Figures 2A–C), observed by staining with fluorogenic probe DCFH-DA. In addition, we have observed HG-induced elevated MDA level (a marker of lipid peroxidation) and sustained depletion of GSH in primary neurons (Figure 2D). All the data suggest a stimulatory effect of HG levels on free radical production.

### Composition of Neuron Metabolites Under HG Culture Conditions

**Figure 3:**
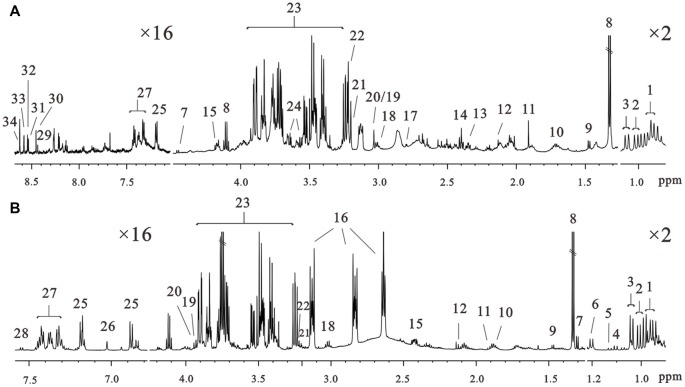
Representative 600 MHz 1D ^1^H nuclear magneticresonance (NMR) spectra of the intracellular extracts **(A)** and culture media **(B)** at 72 h of culture in the control groups. Assignments: 1, leucine; 2, isoleucine; 3, valine; 4, isobutyrate; 5, propylene glycol; 6, 3-hydroxybutyrate; 7, fucose; 8, lactate; 9, alanine; 10, arginine; 11, acetate; 12, methionine; 13, glutamate; 14, succinate; 15, pyroglutamate; 16, N-2-hydroxyethylpiperazine-N-2-ethane sulfonic acid; 17, aspartate; 18, lysine; 19, creatine; 20, phosphocreatine; 21, choline; 22, phosphocholine; 23, glucose; 24, glycerol; 25, tyrosine; 26, τ-methylhistidine; 27, phenylalanine; 28, tryptophan; 29, NAD^+^; 30, formate; 31, ADP; 32, ATP; 33, IMP; 34, AMP.

^1^H NMR spectra of intracellular and extracellular components are shown in Figure 3. The spectral resonances of the metabolites were assigned based on the 600 MHz libraries of Chenomx NMR suite 7.0 (Chenomx, Inc., Edmonton, AB, Canada) and confirmed using a public NMR database (The Human Metabolome Database). Thirty-four unique metabolites were identified and categorized into amino acids and derivatives (isoleucine, leucine, valine, alanine, arginine, methionine, glutamate, pyroglutamate, aspartate, lysine, tyrosine, τ-methylhistidine, phenylalanine, tryptophan), carbohydrates (fucose, glucose), organic compounds (isobutyrate, propylene glycol, 3-hydroxybutyrate, lactate, acetate, succinate, formate, glycerol, creatine, phosphocreatine), choline metabolites (choline, phosphocholine), nucleotides analogs (ADP, ATP, IMP, AMP) and media components. We detected metabolites involved in the TCA cycle and amino acid, fatty acids and membrane metabolism pathways.

### Neuron Metabolites Analysis

**Figure 4:**
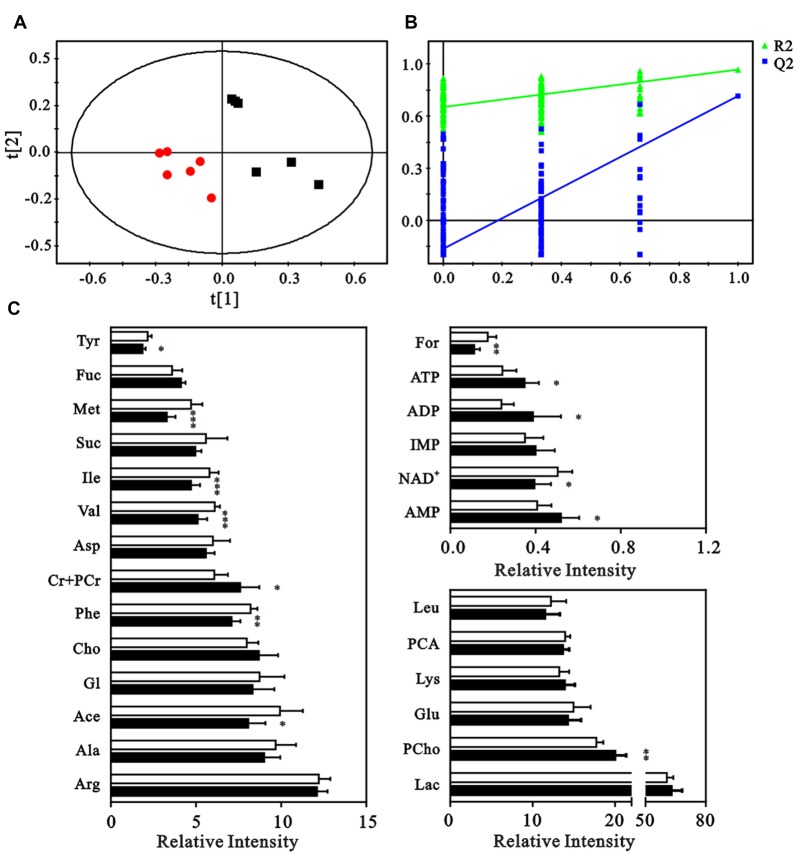
Multivariate analysis and quantitative analysis of intracellular metabolites. **(A)** Partial least-squares discriminate analysis (PLS-DA) score plot to identify the separation between control (black squares) and HG groups (red dots). **(B)** Validation plot by permutation tests (200 cycles). **(C)** The comparison of relative intensities of the intracellular metabolites between control (blank columns) and HG groups (black columns). Data presented as mean ± SD (*n* = 6). Significant level: *p < 0.05, **p < 0.01, ***p < 0.001 vs. control group by independent sample *t*-test.

The neuron metabolite levels measured in the NMR spectra were subjected to partial least-squares discriminate analysis (PLS-DA), which revealed a clear discrimination between the control and HG groups along a PC1 direction. This result suggested an altered metabolic profile under HG exposure (Figure 4A). The validation plot for permutation tests demonstrated that the PLS-DA model constructed for the HG and control groups was robust and credible (*R*^2^Y = 0.925, *Q*^2^ = 0.763, Figure 4B). Metabolite levels were quantified and compared to confirm the metabolic changes (Figure 4C). The HG group exhibited elevated levels of phosphocholine, creatine, phosphocreatine, ATP, ADP, and AMP and decreased formate, nicotinamide adenine dinucleotide (NAD^+^), tyrosine, methionine, acetate, and phenylalanine levels.

### Extracellular Metabolic Footprint Analysis

Longitudinal changes in the culture media of cells exposed to HG and control were measured to determine the kinetic progression of extracellular substances and intracellular metabolites (Supplementary Table S1). Lactate, pyroglutamate, isobutyrate, alanine and propylene glycol were released to the extracellular space under normal physiological glucose concentrations, and isoleucine, lysine, valine, leucine, choline, phosphocholine, 3-hydroxybutyrate, tyrosine, phenylalanine, arginine, acetate, creatine, phosphocreatine, fucose, and tryptophan were absorbed into neuronal cells. However, higher levels of methionine and propylene glycol were detected in the extracellular space after HG exposure, and levels of pyroglutamate were reduced. These results suggest a changed kinetic progression of metabolites across neurons. We observed that neurons absorbed more fucose and τ-methylhistidine from the culture media but less isoleucine vs. controls.

### Correlation Analysis

**Figure 5:**
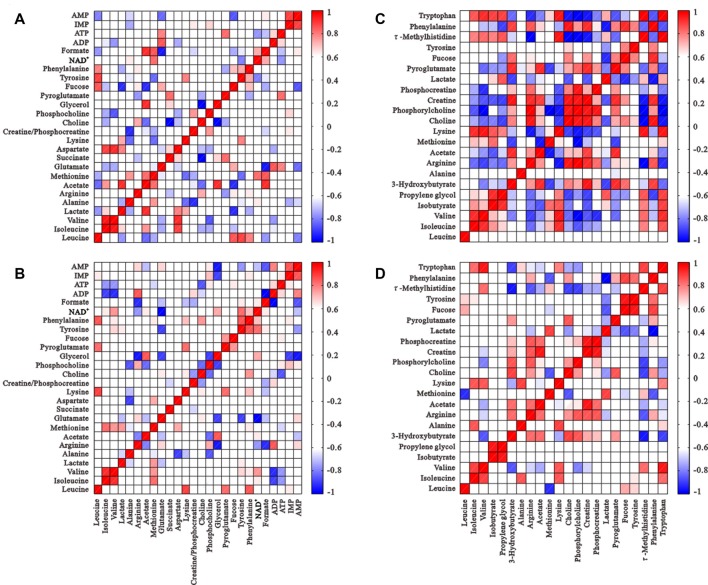
Correlation matrices of metabolite balance: intracellular metabolites of the control group **(A)** and HG group **(B)**; metabolites from media from the control group **(C)** and HG group **(D)**. Red and blue represent positive and negative correlations respectively. The color scale represents Pearson’s correlation coefficients. Coefficients ≥0.70 were considered as statistically significant (*p* < 0.05).

The correlation matrices of intracellular and extracellular metabolites were calculated using Pearson correlation coefficients to gain insight into relationships (Figure 5). Many correlations disappeared or weakened compared to controls, which suggests diminished metabolic coupling under HG challenge. Isoleucine and tyrosine levels positively correlated with valine and phenylalanine, which demonstrates their involvement in the synthesis of succinyl-CoA and fumarate (TCA intermediate products), respectively. However, these correlations disappeared after HG treatment. The correlation of ATP levels with many metabolites, such as phosphocholine, glutamate, formate and NAD^+^ was altered significantly, and isoleucine, tyrosine, valine and phenylalanine were not changed, which suggests alterations in some energy metabolic pathways.

### Metabolic Features of Neurons

**Figure 6:**
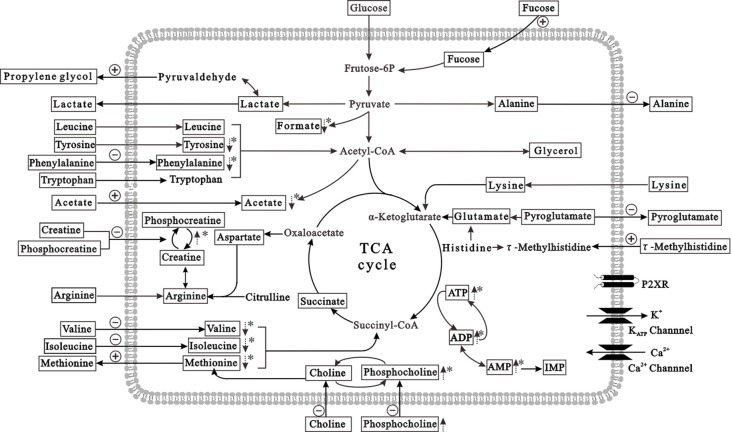
Summary of the prominent metabolites in HG-treated primary neurons (in the intracellular and extracellular compartments). The pathway referenced to the KEGG database and small molecule pathway database (SMPDB). The metabolites with frames are detectable metabolites. The dashed arrows denote significant differences of metabolite levels exposed to HG, compared to controls. The absorption and release of metabolites are labeled with arrows crossing the membrane. The positive and negative signs represent stimulation or inhibition, respectively after HG exposure. *p < 0.05 compared to controls.

Figure 6 presents the metabolic changes in neurons and extracellular compartments in differently treated groups and demonstrates the control and HG responses of the neurons. Lactate, alanine, pyroglutamate and propylene glycol were transported out of neurons under control conditions, and essential amino acids (isoleucine, leucine, valine, arginine, lysine, tyrosine, phenylalanine, tryptophan and methionine), organic acids (acetate, creatine, phosphocreatine, choline and phosphocholine), and carbohydrates (fructose) were absorbed into neurons. However, isoleucine absorption was reduced after HG exposure for 72 h, and alanine and pyroglutamate release was diminished. Propylene glycol and methionine release was greater than controls. The metabolic processes of intracellular and extracellular compartments presented a holistic picture of the disturbed metabolic features of neurons after HG exposure.

### Determination in Adenosine Phosphate Levels

**Figure 7:**
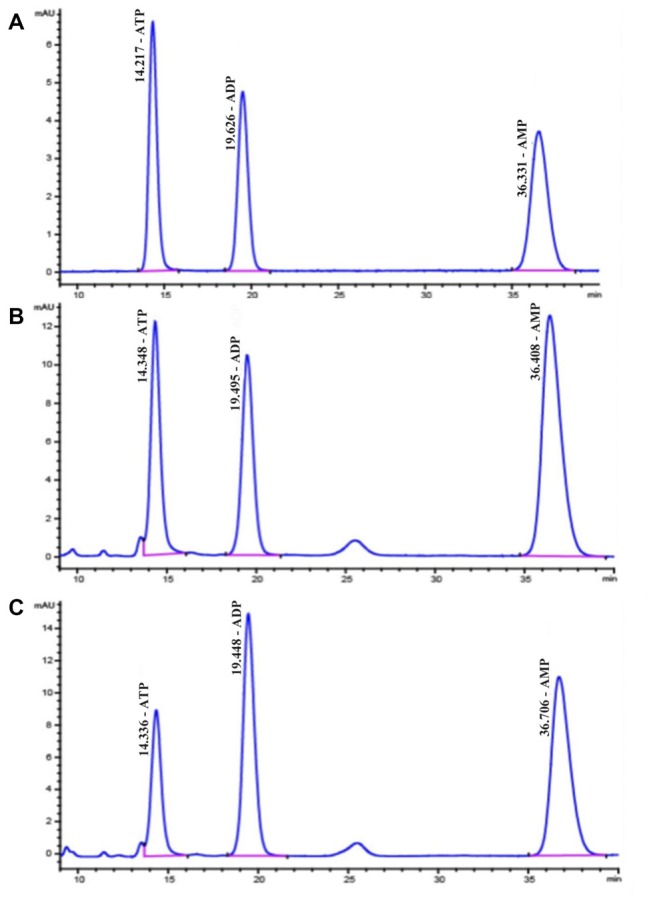
High performance liquid chromatography (HPLC) chromatograms of standard substances of adenine nucleotides **(A)** in hippocampal extracts from diabetic 15-week **(B)** and the control 15-week rats **(C)** In **(A)**, the retention times are as follows: ATP, 14.2 min; ADP, 19.6 min; AMP, 36.3 min.

**Figure 8:**
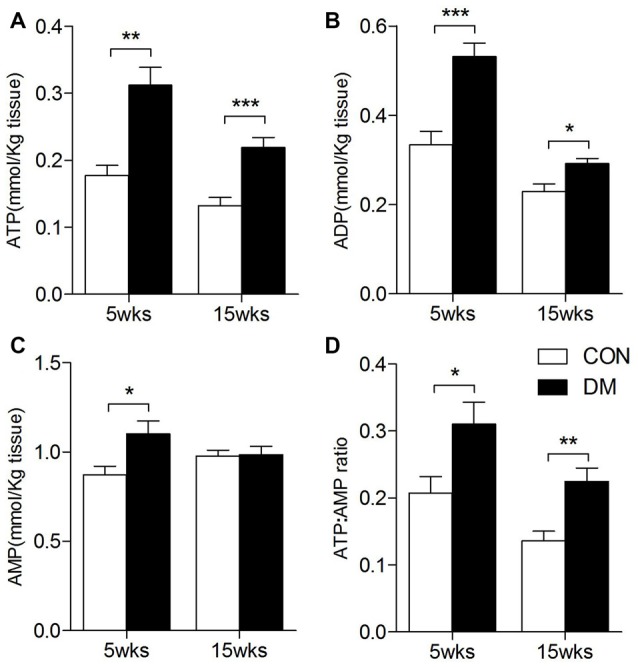
ATP, ADP, AMP levels in the hippocampal extracts of 5- and 15-week diabetes mellitus (DM) rats using HPLC. Data presented as mean ± SD (*n* = 6). Significant level: *p < 0.05, **p < 0.01, ***p < 0.001 vs. age-matched control rats by independent sample *t*-test. **(A)**, ATP levels; **(B)**, ADP levels; **(C)**, AMP levels; **(D)**, ATP/AMP ratio.

ATP, ADP and AMP levels in hippocampal extracts of 5- and 15-week STZ-induced diabetic rats and age-matched control rats were analyzed using HPLC technology and an external standard method. Figure 7 shows representative chromatograms obtained from standard and hippocampal extracts from diabetic and control rats. The retention times of ATP, ADP and AMP were 14.2, 19.6 and 36.3 min, respectively. Figure 8 shows a comparison of adenosine metabolite concentrations in hippocampal extracts from different groups. Significant elevations in ATP and ADP concentrations were observed in hippocampal extracts from 5- and 15-week diabetic rats compared to control rats. ATP/AMP ratios were also markedly increased by 50%–100% at both time points. HPLC data of the diabetic hippocampal extracts were consistent with the NMR data of *in vitro* cultured neurons, which suggests a specific elevation of ATP or energy metabolic levels in hyperglycemic conditions.

## Discussion

Neurons in the central nervous system (CNS) play a fundamental role in the transmitting of information via electrical and chemical signals (Andersen, 2004; Hirokawa et al., 2010; Liu et al., 2014). Cognitive impairment resulting from neuronal apoptosis is a risk factor in diabetic patients (Roriz-Filho et al., 2009; Liu et al., 2013). The present study demonstrated HG-induced apoptosis in primary neurons, which was characterized by significant decreases in viability and increases in oxidative stress compared to controls. Neuron-specific metabolic alterations were investigated using NMR-based metabonomic technologies with HPLC as a supplement. Our characterization of the metabolic changes derived from *in vitro* and *in vivo* experiments will be useful to understand the impact of hyperglycemia on cognitive function.

### Neuron-Specific Metabolic Alterations

Intracellular metabolic changes are a direct response to external stimuli, and changes in extracellular substances reflect changes in cellular metabolism or function. Our primary cell study that mimicked diabetic conditions detected decreased absorption of many essential amino acids, e.g., isoleucine, valine and phenylalanine, from the culture media and decreased levels in neurons, which suggests changes in the metabolism of some amino acids. Correlation analyses identified that the inherent positive correlation in the levels of the amino acids involved in the synthesis of succinyl-CoA and fumarate diminished after HG treatment. Therefore, variations in TCA cycle may be one of the major metabolic features in local neurons under diabetic conditions.

NMR spectra analysis of neurons revealed significant increases in intracellular ATP and ADP levels after HG exposure, which contrasts the alterations in astrocytes observed in our previous study (Wang et al., 2018), which suggests altered energy levels specific to neurons. HPLC technology was further used to determine the levels of ATP, ADP, and AMP in hippocampal extracts from STZ-induced diabetic rats. Similarly, there were also significant increases in the levels of ATP, ADP and ATP/AMP ratios in 5-week (short-term) and 15-week (long-term) diabetic rats compared to age-matched controls. These results suggest neuron-specific energy metabolism alterations in diabetic hippocampus during the entire pathogenic process.

ATP is a substance that stores energy *in vivo*, but recent studies demonstrated that extracellular ATP was also an important neurotransmitter. Hypoxia and the decrease in cell vitality in the CNS promotes an increase in ATP contents (Eltzschig and Carmeliet, 2011). ATP also leads to damage to surrounding cells (Rissiek et al., 2015). ATP selectively stimulates P2 purine receptors, such as the ionic receptor P2X7 (Burnstock, 2016), which eventually leads to cell apoptosis (Rodrigues et al., 2005; Di Virgilio et al., 2009; Idzko et al., 2014). However, other reports also revealed that ATP-driven modulation in rat neurons relied on the K_ATP_ channel (Levin et al., 1999), Ca^2+^ influx (Abeti and Abramov, 2015; Liu et al., 2017), or NO production (Lowe et al., 2013). Therefore, the causal link between ATP and cognition impairment and the molecular mechanisms involved must be further examined in the future.

The present study identified elevated ATP levels in HG-treated neurons, but not astrocytes, which suggests that the increased energy biosynthesis in hippocampal regions may arise from neuronal metabolism. We confirmed altered TCA cycle in neurons after HG treatment, which is consistent with our previous study using ^13^C-labeled lactate and acetate as substrates. we identified an increased rate of TCA cycle in neurons of *db/db* mice with cognitive dysfunction (Zheng et al., 2017b). All of the data indicate that enhanced ATP production in the brain may be the result of an increased TCA cycle rate in local neurons, which provide more options for the development of drugs to intervene in the CNS complications of diabetes in the future.

### Neuron-Astrocyte Metabolic Coupling

The neuron-astrocyte metabolic link is appealing because it underscores the importance of the functional connection of these two cell types to maintain brain functions, including cognitive ability (Supplementary Figure S2). The neuron-astrocyte lactate shuttle (ANLS) hypothesis proposes that glucose is predominantly metabolized to lactate in astrocytes, transported to the extracellular compartment via monocarboxylate transporters (MCT1, MCT4), for use by surrounding neurons (MCT2) in physiological states (Pellerin and Magistretti, 1994). Our studies confirmed that astrocyte, but not neuron, produced more lactate in hyperglycemic conditions, which was similar to the ANLS phenomena in physiological conditions. Lactate was considered a signaling molecule that coordinates astrocyte and neuron function (Chih et al., 2001; Medina and Tabernero, 2005). The present data contribute to our understanding of the effects of elevated lactate levels in hippocampal extracts that we identified previously (Zheng et al., 2017a). We also found that acetate, which exhibits a similar transport process to lactate, was also transferred from astrocyte (Wang et al., 2018).

The ^1^H NMR spectra of primary neuronal cells confirmed that certain essential amino acids, e.g., valine, leucine, isoleucine, phenylalanine, methionine and tyrosine, were absorbed and used by neurons and astrocytes under physiological conditions (Supplementary Figure S2). However, only astrocyte increased the consumption of essential amino acids for biosynthesis, including phenylalanine, valine, isoleucine, leucine, and tyrosine, under hyperglycemic conditions. We also found that astrocyte, but not neuron, also produced osmotic regulators, such as taurine and myo-inositol. These cell-specific metabolic changes provide us more detailed information than our previous animal experiments (Zheng et al., 2016, 2017c) and help us further elucidate the pathogenesis of brain dysfunction in diabetes.

In summary, our study revealed specific information of metabolite levels and transport process related to neurons in diabetic conditions, i.e., ATP, TCA cycle, as well as transport features of lactate and acetate, which were different from astrocytes. The results also identified that both glycolysis and TCA cycle, were changed in cell-specific manner. Our future work will be focused on proving the causal link between the ATP, lactate levels and diabetes associated cognitive impairment.

## Author Contributions

HG and LZ contributed to experimental design and writing of the manuscript. DW, YZ, HZ and MD contributed to animal experiment data acquisition. CL and HZ contributed to data analysis, result interpretation. All authors have read and approved the final manuscript.

## Conflict of Interest Statement

The authors declare that the research was conducted in the absence of any commercial or financial relationships that could be construed as a potential conflict of interest.
