# Supplementary material for: Characteristic Metabolic Alterations Identified in Primary Neurons Under High Glucose Exposure
Source: Front Cell Neurosci. 2018 Jul 17;12:207. doi: 10.3389/fncel.2018.00207 (PMC6056731; doi:10.3389/fncel.2018.00207)
Supplement: Supplementary file 1 [file Presentation_1.pdf]

Supplementary materials

Table S1. Dynamic changes in metablites after 72 h high glucose circumstances.

Metabolites	CON levels	HG levels	Influx/efflux	P values
Leucine	-5.74 ± 1.03	-5.52 ± 0.91	Influx	0.69
Isoleucine	-10.13 ± 2.09	-7.84 ± 0.69	Influx	0.01
Valine	-7.54 ± 0.53	-5.38 ± 0.73	Influx	0.04
Isobutyrate	1.67 ± 0.16	1.82 ± 0.07	Efflux	0.36
Propylene glycol	0.72 ± 0.13	0.94 ± 0.10	Efflux	0.06
3-Hydroxybutyrate	-3.55 ± 0.82	-1.87 ± 0.11	Influx	0.43
Alanine	0.95 ± 0.09	0.60 ± 0.09	Efflux	0.00
Arginine	-1.90 ± 1.26	-1.26 ± 0.21	Influx	0.26
Acetate	-2.07 ± 0.13	-2.68 ± 0.13	Influx	0.04
Methionine	-0.14 ± 0.34	-1.11 ± 0.70	Efflux	0.04
O-Acetylcholine	-1.94 ± 0.32	-1.60 ± 0.21	Efflux	0.35
Lysine	-8.60 ± 5.03	-9.23 ± 1.40	Influx	0.10
Choline	-4.62 ± 0.18	-4.22 ± 0.30	Influx	0.05
PCho	-2.95 ± 0.45	-1.90 ± 0.18	Influx	0.05
Creatine	-1.35 ± 0.42	-0.68 ± 0.11	Influx	0.00
Creatine Phosphate	-1.20 ± 0.47	-0.15 ± 0.12	Influx	0.00
Lactate	36.34 ± 5.14	36.77 ± 5.00	Efflux	0.70
Pyroglutamate	2.54 ± 0.20	0.30 ± 0.08	Efflux	0.00
Fucose	-0.32 ± 0.05	-0.45 ± 0.05	Influx	0.00
Calactose	-0.18 ± 0.04	-0.16 ± 0.04	Influx	0.71
Tyrosine	-2.30 ± 0.24	-2.18 ± 0.16	Influx	0.19
τ-methylhistidine	-0.30 ± 0.04	-0.25 ± 0.03	Influx	0.04
Phenylalanine	-2.71 ± 0.31	-2.08 ± 0.17	Influx	0.02
Tryptophan	-0.09 ± 0.02	-0.16 ± 0.01	Influx	0.56
Pyridoxine	-0.72 ± 0.13	-0.23 ± 0.02	Influx	0.04

Data were obtained by subtracting the levels measured in the medium at 72 h of culture from the levels measured at time pre-dose. Positive values represent efflux, negative values represent influx. Values represent the mean ± SD of 6 samples in each group. Statistical comparisons were performed by independent sample t-test.

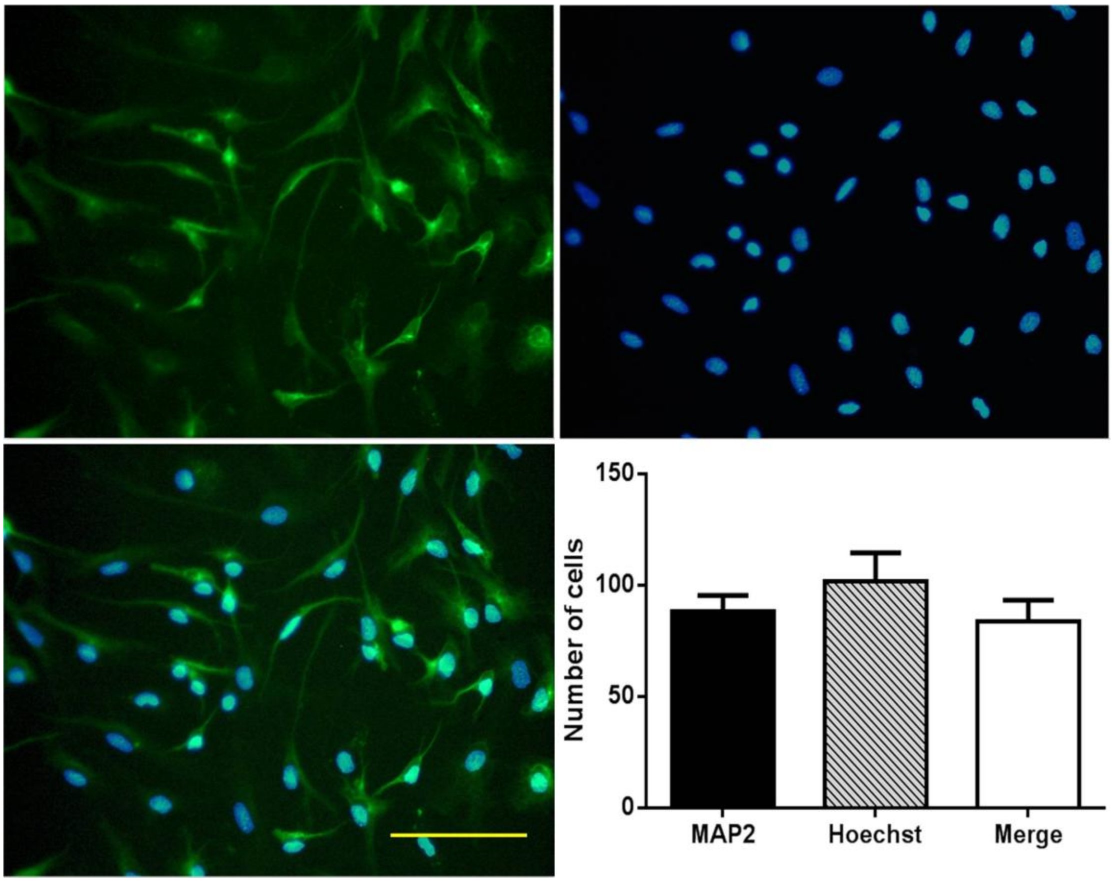

Figure S1. Immunofluorescence micrographs of MAP2 (green) positive neurons and nuclei were counterstained by Hoechst 33342 (blue), revealed neuron purity > 90%. (Scale bar = 500 μm).

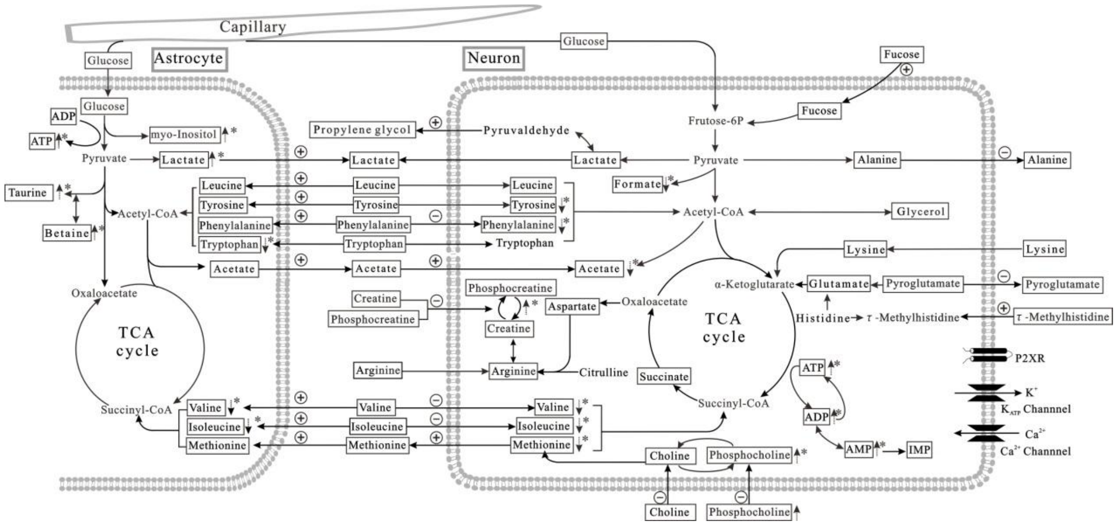

Figure S2. Overviewed the metabolic coupling of the identified metabolies between neurons and astrocytes. The pathway referenced to the KEGG database and small molecule pathway database (SMPDB). The dashed arrows means significant differences exposured to HG, compared to controls. The absorption and release transports of metabolites are directed with arrows acrossing membrane. The positive and negative signs represent the tendency of activity and inhibition, respectively, under HG circumstance. *$P < 0.05$ compared to controls.
